# Rehabilitation Outcomes among Frail Older Adults in the United States

**DOI:** 10.3390/ijerph191711021

**Published:** 2022-09-03

**Authors:** Jason R. Falvey, Joanna Z. Ye, Elizabeth A. Parker, Brock A. Beamer, Odessa Addison

**Affiliations:** 1 Department of Physical Therapy and Rehabilitation Science, University of Maryland School of Medicine, Baltimore, MD 21201, USA; 2 Department of Epidemiology and Public Health, University of Maryland School of Medicine, Baltimore, MD 21201, USA; 3 Department of Veterans Affairs and Veterans Affairs Medical Center Baltimore, Geriatric Research, Education and Clinical Center (GRECC), Baltimore, MD 21201, USA

**Keywords:** frailty, older adults, rehabilitation, physical therapy

## Abstract

Background: Current rehabilitation care paradigms are not well aligned with the needs of frail older adults, but the resultant impact on rehabilitation outcomes is unclear. Understanding how frailty may impact rehabilitation outcomes, and understanding some of the underlying mechanisms, may help inform payment policy changes. Design: This study was a cross-sectional analysis of data from Round 5 of the National Health and Aging and Trends Study (NHATS). We identified older adults who had completed one or more episodes of rehabilitation care and used a validated 5-item NHATS Fried Frailty scale to categorize patients as frail (3/5 or more) or non-frail (≤2/5). We then evaluated the association between frailty status and three key patient outcomes: (1) achievement of rehabilitation goals, (2) functional improvement during rehabilitation episodes, and (3) discontinuation of therapy after exhausting insurance benefits. Lastly, we used multivariable, survey-weighted logistic regression models to estimate adjusted relationships between frailty and rehabilitation outcomes. Results: An estimated 5.6 million survey-weighted older adults in the United States (95% CI 5.1 to 6.0 million) completed an episode of rehabilitation in the past year, an estimated 1,271,290 (95% CI 921,758 to 1,620,822; weighted: 22.8%) of whom were frail. Frail rehabilitation recipients were generally older, had a greater comorbidity burden, and had a higher prevalence of dementia. In adjusted models, frailty was associated with poorer functional outcomes, a lower probability of meeting rehabilitation goals and a greater likelihood of exhausting rehabilitation insurance benefits. Conclusions: Exercise is a well-supported intervention for the management of frailty, but our results suggest that frail older adults are not getting the volume or intensity of rehabilitation treatment needed to maximally improve outcomes—in part due to limited payer coverage of rehabilitation services in the United States.

## 1. Introduction 

An estimated 15% of all older adults in the United States are frail [1]. Even among otherwise healthy older adults, frailty is a powerful antecedent for adverse health outcomes. Frailty is independent from, but confers increased vulnerability to, disability among older adults, and is associated with elevated risk for institutionalization and death [2,3]. Among the tested interventions to help mitigate the impact of frailty on older adults, tailored and well-supervised exercise interventions in well-controlled clinical trial environments have shown promise in improving function. However, the translation of these exercise paradigms to real-world rehabilitation settings, with less structure and supervision, has been poor [4,5]. 

Among the potential mechanisms for poor translation of exercise research into clinical rehabilitation settings, is the poor alignment of insurance payment paradigms in the United States with care needs for frail older adults. Rehabilitation in a hospital, skilled nursing facility, or inpatient rehabilitation facility is often delivered over a short length of time (generally, <20 days), which might not be enough to address the long-standing deficits related to frailty [6]. Similarly, home-based rehabilitation is generally delivered in short care episodes (30–60 days) and can only be provided under Medicare benefits for older adults if a patient is homebound. In outpatient rehabilitation models, frail older adults often must travel to a clinic before participating in restorative treatments, a daunting challenge experienced by patients for whom walking even short distances is exhausting. Lastly, explicit Medicare or other payer policies such as payment caps or cost-sharing related to rehabilitation service use [7] may disproportionately affect frail older adults who typically require a high volume of therapy delivered over a longer period of time to realize meaningful functional gains [5]. Yet, there is a paucity of literature looking at patient-centered rehabilitation outcomes for frail older adults and even less exploring the adequacy of insurance coverage for rehabilitation. Understanding the impact of frailty on rehabilitation patient-centered outcomes, and the adequacy of rehabilitation coverage for frail older adults may inform important changes in payment and practice. 

Thus, the purpose of this study is to evaluate the impact of frailty on patient-reported rehabilitation outcomes among older adults, and secondarily assess whether frail older adults are more likely to exhaust their insurance rehabilitation benefits during an episode of care. To answer these important questions, we leveraged the National Health and Aging Trends Study (NHATS), a nationally representative sample of Medicare beneficiaries aged 65 and older in the United States. Our hypothesis was that frail older adults would be more likely to report poor rehabilitation outcomes and have a greater likelihood of exhausting insurance benefits. 

## 2. Methods

This cohort study used data from the 5th wave (Round 5) of the NHATS survey. The details of this complex, nationally representative survey of older adults in the United States have been published elsewhere [8]. Briefly, the NHATS cohort was drawn from the records of US older adults in Medicare enrollment files in 2011 and replenished in 2015 (Round 5) to maintain nationally representative estimates of late-life disability among older adults, with oversampling of those who identified as non-Hispanic Black or who were older than 85. Annual in-person interviews were conducted with participants, collecting data on important domains such as physical function, social functioning, socioeconomic status, and health conditions. During Round 5, questions on rehabilitation utilization, goals, and outcomes were added to the survey battery for the first time. The Johns Hopkins University Institutional Review Board (IRB) approved the NHATS protocol, and all participants provided informed consent. The use of publicly available NHATS data for this analysis is considered exempt, non-human subjects research by the University of Maryland Institutional Review Board. 

### 2.1. Identification of Rehabilitation Participants and Outcomes

During the Round 5 annual interview, participants were asked: “In the last year, have you received any rehabilitation services?” Participants who responded affirmatively were coded as rehabilitation participants for our analysis. We additionally identified the setting in which rehabilitation was received (inpatient, home-based, or outpatient), whether rehabilitation was being received after surgery, what was the main medical condition for which rehabilitation was received, and what patient-identified concerns were they hoping to address. 

### 2.2. Identification of Frailty

Frailty among rehabilitation participants was identified using a modified Fried frailty score (0 to 5 scale with key domains of exhaustion, low physical activity, weakness, slowness, and shrinking) developed in NHATS data by Bandeen-Roche and colleagues [1]. Participants were considered frail if they met three or more of the criteria for frailty, and non-frail if they had two or fewer. 

### 2.3. Rehabilitation Outcomes

Using self-reported data from the rehabilitation module of the NHATS survey, we captured whether the patients met all or most of their goals when rehabilitation services ended (yes/no), whether their functioning or ability improved while receiving rehabilitation as compared to worsened function or unchanged (categorized as improved/not improved), and whether they had met the limits of their insurance coverage when rehabilitation services ended (yes/no). Participants who refused to answer or responded that they did not know the answer to the questions were categorized as missing (goals: *n* = 3; functioning: *n* = 4, and insurance *n* = 158). The text of the questions is provided in the Appendix A Table A1. 

### 2.4. Demographic Characteristics

To characterize the sample of rehabilitation users, we extracted age, sex, self-reported race (for descriptive purposes), chronic health conditions, dementia status (possible or probable as defined by prior NHATS work), history of falls, hospitalizations in the last year, and whether the participant was in a marriage or partnership. We also included an assessment of food insecurity using a previously validated measure to assess the difference in nutritional status among frail and non-frail rehabilitation users [9]. 

### 2.5. Selection of the Analytic Sample

**Figure A1:**
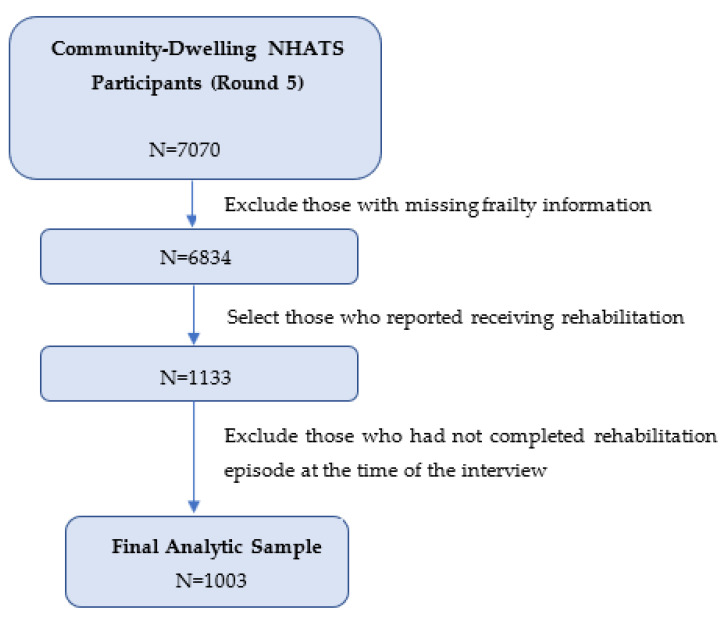
Selection of the Analytic Sample. **Legend:** Flow diagram depicting selection of the analytic sample from the National Health and Aging Trends Study, Round 5 data.

In round 5, we included only those participants in NHATS who were community-dwelling (*n* = 7070), excluding those living in nursing homes or other institutional settings. We then excluded a small number (*n* = 236) of participants with missing frailty data that precluded accurate classification (e.g., those with missing data for >3/5 frailty criteria). Our final analytic sample consisted of the 1003 older adults who had completed an episode of rehabilitation care in the year prior to the NHATS interview (Figure A1). 

### 2.6. Statistical Approach

Unweighted demographic and clinical characteristics of the population were recorded, as well as characteristics of rehabilitation episodes stratified by participant frailty status. Accounting for the strata and clustering of the NHATS survey and applying analytic weights that help adjust for differential non-response, we then generated a nationally representative estimate of frailty among rehabilitation users in the United States. 

Next, we modeled the association between frailty and our three rehabilitation outcomes (goals not met, function not improved, and insurance benefits exhausted) using multivariable logistic regression. Multivariable models were adjusted for age, sex, surgical history, dementia classification, a count of activities of daily living for which a person need help from another person, and the presence of any of the following chronic health conditions: prior myocardial infarction, heart disease, hypertension, arthritis, osteoporosis, diabetes mellitus, and lung disease. The resultant adjusted odds ratios (aOR) and 95% confidence intervals (CI) represent the relative odds of frail rehabilitation participants experiencing the outcome as compared to a non-frail participant. 

To account for the complex survey design, all multivariable models included analytic weights, strata, and clustering parameters specific to the 2015 NHATS cohort. In all cases, statistical significance was defined as a two-sided *p*-value < 0.05; because this study was exploratory, we did not adjust for multiple tests. Data analysis was conducted from November 2021 through April 2022. All analyses were performed in SAS 9.4 (SAS Institute, Cary, NC, USA).

## 3. Results

The primary analysis included 1003 rehabilitation episodes, representing an estimated 5,577,972 survey-weighted older adults in the United States (95% CI 5,152,434 to 6,003,510). Of those, 292 were classified as frail (estimated 1, 271,290 older adults, 95% CI 921,758 to 1,620,822; weighted percentage of rehabilitation users: 22.8%). 

Relative to non-frail participants in our sample, frail rehabilitation participants were older, more likely to be female sex, had greater comorbidity burden, and higher prevalence of dementia (Table 1). Food insecurity was also notably higher among the frail rehabilitation users (*n* = 47/289, 16.3%) as compared to non-frail (*n* = 37/698, 5.3%). Lastly, frail patients more commonly received rehabilitation in inpatient settings or home settings versus outpatient settings as compared to non-frail rehabilitation participants (Table 1). Additional descriptive analyses suggest the tasks older rehabilitation users wanted to improve differed between frail and non-frail participants. Frail older adults were more likely to seek improvements in ADLs and other basic mobility tasks such as walking inside as compared to higher-level activities such as working (Appendix A Table A2, Table A3 and Table A4). 

**Figure 1:**
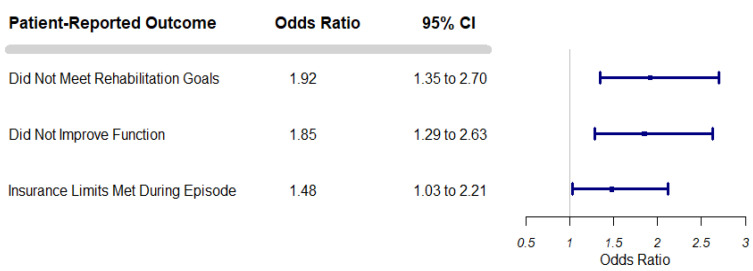
Rehabilitation Outcomes for Frail Older Adults as Compared to Non Frail Older Adults. **Legend:** Adjusted odds ratios and 95% confidence intervals for self-reported rehabilitation outcomes among frail older adults are depicted in the figure, with non-frail older adults as the reference. Data were drawn from Round 5 of the National Health and Aging Trends Study. Models were adjusted for age, sex, whether or not rehabilitation was following a surgical procedure, prior ADL disability, dementia classification, and the self-reported presence of any of the following chronic conditions: prior myocardial infarction, heart disease, hypertension, arthritis, osteoporosis, diabetes mellitus, and lung disease. Additionally, models accounted for the complex design of the NHATS survey.

Rehabilitation outcomes differed across frail and non-frail older adults. Frail older adults were more likely to report they did not meet rehabilitation goals compared to non-frail older adults, more likely to report that they did not make improvements in function and were more likely to report that they exhausted their insurance benefits by the time therapy ended (Table 2)—these outcome disparities persisted in adjusted models (Figure 1). 

## 4. Discussion

In this nationally representative study of older rehabilitation users, we observed that frailty was associated with poorer outcomes, a lower probability of meeting even modest rehabilitation goals and a greater likelihood of exhausting rehabilitation insurance benefits. These disparities persisted after adjustment for other geriatric vulnerabilities and medical complexity. This is concerning given our data also suggests nearly 1 in 4 older adults seeking rehabilitation in the US healthcare system are frail, with substantial increases in the prevalence of frailty for those over 80 years of age. These findings are novel and have significant implications for several ongoing payment reforms and the design of insurance benefits related to rehabilitation. 

Prior work evaluating functional outcomes after disabling hospitalizations shows that frailty is associated with a poorer recovery in activities of daily living (ADLs) and instrumental ADLs (IADLs) [10,11]. A common limitation of these studies is a lack of information on restorative care—our study suggests one potential reason why frail older adults have poorer functional recovery is the exhaustion of rehabilitation insurance benefits before functional goals were reached. Additionally, there are several plausible biological mechanisms for why frail older adults may not respond as robustly to rehabilitation care that could be explored in future studies. First, food insecurity was notably more common among frail older rehabilitation users in our study—important because this may represent a proxy of poor dietary quality, which is associated with a loss of muscle mass and strength [12]. Routine assessment of food insecurity, dietary intake, and frailty are likely not occurring across all rehabilitation settings—a potentially modifiable factor to improve rehabilitation outcomes [13]. Specifically, frail older adults who have poor protein intake or low dietary quality may not be able to respond as robustly to rehabilitation [13,14]. 

Exercise is considered a first-line treatment for the management of frailty, and at least one study has shown multicomponent exercise programs undertaken over a period of 24 weeks have generally shown to be most effective at addressing functional impairments [15]. Yet, typical rehabilitation in the United States is delivered over much shorter periods and at lower intensities than shown in clinical trials, which may leave older adults with unaddressed vulnerability to disability and costly nursing home admissions. Ensuring older adults have access to rehabilitation delivered at the appropriate dose—intensity, frequency, and duration—is thus paramount in optimal frailty management and may require a re-evaluation of the intensity and duration of contemporary rehabilitation programs. The need to restructure care is especially acute for the oldest-old in the United States, a growing subgroup in the United States that is increasingly vulnerable to becoming homebound or requiring care in a nursing home. 

Rehabilitation services could play a vital role in promoting high-quality aging in place among frail older adults. Unfortunately, our findings suggest current payer policies related to rehabilitation may be inadequate for the frail population. First, several Medicare policies cap receipt of outpatient rehabilitation services through the use of somewhat arbitrary cost thresholds—thresholds that Medicare explicitly indicates should be exceeded sparingly in billing guidance [7]. Another example is extended stays in skilled nursing facilities, which require cost sharing by patients after 20 days—thereby disadvantaging frail patients who may have prolonged courses of recovery and could result in premature discharge prior to goals being met. Third, Medicare Advantage payers often cover rehabilitation services but 1 in 3 plans require pre-authorizations or restrict visit counts which could delay or deter frail older adults from receiving high-quality care [16]. Additionally, frail older adults in our study were more than threefold as likely to report Medicaid coverage, which typically is only available to those living below the poverty line. This suggests unique intersectional vulnerabilities between age, poverty, and frailty that may need to be addressed in future payment reforms. These reforms go beyond simply extending therapy coverage, but also expanding access to other benefits, such as nutritional supports, that may support better responses to tailored rehabilitation interventions. 

Our study has several key strengths and some limitations that need to be acknowledged. Major strengths include the use of a large nationally representative dataset capturing both a well-validated frailty phenotype and several important domains of rehabilitation use and outcomes for community-dwelling older adults. Our study was limited by an inability to determine which rehabilitation service was predominantly provided (physical, occupational, or speech therapy)—it is unclear whether frail older adults are receiving services from those disciplines in different ways. We also did not know the specific diagnoses for which PT was sought. Our study was also cross-sectional, which did not allow us to evaluate causal or temporal relationships between frailty and the observed vulnerabilities—later studies may help better untangle the mechanisms contributing to frailty among this population to better guide rehabilitation interventions. 

## 5. Conclusions

Rehabilitation therapy is a critical service for older adults in the United States and may be particularly important for those with frailty—who are more likely to be over 80 years of age, socioeconomically disadvantaged, and experiencing food insecurity as compared to their peers. Frail older adults have poorer outcomes when receiving rehabilitation care and are more likely to report stopping therapy after exhausting insurance benefits. Exercise is a well-supported intervention for the management of frailty, but our results suggest that frail older adults may not be getting the volume or intensity of rehabilitation treatment needed to maximally improve outcomes—in part due to limited payer coverage of rehabilitation services in the United States. Both changes in clinical processes and payer policies are likely needed to mitigate frailty-related outcome disparities for older rehabilitation users.

## Figures and Tables

**Table 1:** Demographic Characteristics of Older Adults by Frailty Status Using the National Health and Aging Trends Study (NHATS), United States, 2015.

Characteristic	NHATS Total (*n* = 1003)	Frail (*n* = 292)	Non-Frail (*n* = 711)
**Sample *n***	1003	292	711
**Survey-weighted *n***	5,577,972	1,271,290	4,306,682
**Age Category, *n* (%)**			
65–69	120 (12.0)	23 (7.9)	97 (13.6)
70–74	243 (24.2)	49 (16.8)	194 (27.3)
75–79	205 (20.4)	42 (14.4)	163 (22.9)
80–84	208 (20.7)	68 (23.3)	140 (19.7)
85–89	137 (13.7)	60 (20.6)	77 (10.8)
>90	90 (9.0)	50 (5.0)	40 (5.6)
**Male sex, *n* (%)**	365 (36.4)	95 (32.5)	270 (38.0)
**Race/Ethnicity, *n* (%)**			
Non-Hispanic White	745 (74.3)	177 (60.6)	568 (79.9)
Non-Hispanic Black	167 (16.7)	76 (26.0)	91 (9.1)
Hispanic	47 (4.7)	20 (6.9)	27 (3.8)
Other	16 (1.6)	3 (1.0)	13 (1.8)
**Marital Status, *n* (%)**			
Married or living with partner	516 (51.5)	111 (38.0)	405 (57.0)
Not married or living with a partner	487 (48.6)	181 (62.0)	306 (43.04)
**Comorbidities, *n* (%)**			
Myocardial infarct	110 (11.0)	40 (13.7)	70 (9.9)
Heart disease	269 (26.8)	111 (38.0)	158 (22.2)
Hypertension	731 (72.9)	237 (81.2)	494 (69.5)
Arthritis	731 (72.9)	239 (81.9)	492 (69.2)
Osteoporosis	310 (30.9)	104 (35.6)	206 (29.0)
Diabetes	266 (26.5)	102 (34.9)	164 (23.7)
Lung disease	218 (21.7)	83 (28.4)	135 (19.0)
**Dementia, *n* (%)**			
Possible	82 (8.2)	37 (12.7)	45 (6.3)
Probable	108 (10.8)	73 (25.0)	35 (4.9)
**Fell in last month, *n* (%)**	170 (17.0)	66 (22.6)	104 (14.6)
**Hospitalized in past year, *n* (%)**	485 (48.4)	188 (64.4)	297 (41.8)
**Medicaid, *n* (%)**	115 (12.1)	65 (24.7)	50 (7.3)
**Received rehab services, *n* (%)**			
Overnight hospital	319 (31.8)	120 (41.1)	199 (28.0)
Outpatient	640 (63.8)	112 (38.4)	528 (74.3)
Home	378 (37.7)	172 (58.9)	206 (29.0)
Somewhere else	70 (7.0)	21 (7.2)	49 (6.9)
**Food insecurity, *n* (%)**	84 (8.5)	47 (16.3)	37 (5.3)
**Post-surgery rehabilitation, *n* (%)**	344 (34.4)	89 (30.5)	255 (36.0)

Missing variable counts for the variables in the table are as follows: Medicaid coverage (*n* = 53), food insecurity (*n* = 16), rehab surgery (*n* = 3).

**Table 2:** Rehabilitation Outcomes by Frailty Status Using the National Health and Aging Trends Study (NHATS), United States, 2015.

Outcome	NHATS Total (*n* = 1003)	Frail (*n* = 292)	Non-Frail (*n* = 711)
**Did not meet goals, *n* (%)**	258 (26.1)	108 (38.4)	150 (21.2)
**Did not improve function during rehab, *n* (%)**	293 (29.2)	137 (47.1)	156 (21.9)
**Did not improve function after rehab, *n* (%)**	544 (54.4)	200 (68.7)	344 (48.5)
**Exhausted insurance benefits, *n* (%)**	322 (38.1)	120 (50.2)	202 (33.3)

Missing variable counts for the variables in the table are as follows: did not meet goals (*n* = 14), did not improve function during (*n* = 1), did not improve function after (*n* = 3), exhausted insurance benefits (*n* = 158).

## Data Availability

Data from National Health and Aging Trends Study (NHATS) is sponsored by the National Institute on Aging (grant number NIA U01AG32947) and was conducted by the Johns Hopkins University. This data is publicly available at https://nhats.org/researcher. Accessed 25 September 2021.

## Appendix A

**Table A1:** Rehabilitation Outcomes Assessed in the National Health and Aging Trends Study (NHATS), United States, 2015.

Problems	
Goal Attainment	When your rehab services ended, had you met all or most of your goals?
Functional Improvement	While you were receiving rehab services in the last year, did your functioning and ability to do activities improve, get worse, or stay about the same?
Insurance Exhaustion	When your rehab services ended, had you met the limit of your insurance coverage?

**Table A2:** Reasons for Seeking Rehabilitation Services by Frailty Status Using the National Health and Aging Trends Study (NHATS), United States, 2015: Problems.

Problems	NHATS Total (*n* = 1003)	Frail (*n* = 292)	Non-Frail (*n* = 711)
Improve strength, *n* (%)	551 (54.9)	187 (64.0)	364 (51.2)
Improve movement/range of motion, *n* (%)	597 (59.5)	175 (59.9)	422 (59.4)
Improve pain level, *n* (%)	346 (34.5)	89 (30.5)	257 (36.2)
Improve balance/coordination, *n* (%)	351 (35.0)	135 (46.2)	216 (30.4)
Improve problems with falls, *n* (%)	132 (13.2)	64 (21.9)	68 (51.5)

**Table A3:** Reasons for Seeking Rehabilitation Services by Frailty Status Using the National Health and Aging Trends Study (NHATS), United States, 2015: Mobility.

Mobility	NHATS Total (*n* = 1003)	Frail (*n* = 292)	Non-Frail (*n* = 711)
Improve walking inside home, *n* (%)	430 (42.9)	170 (58.2)	260 (36.6)
Improve walking distance outside, *n* (%)	442 (44.1)	114 (39.0)	328 (46.1)
Improve climbing stairs, *n* (%)	309 (30.8)	94 (32.2)	215 (30.2)
Improve leaving home outside, *n* (%)	271 (27.0)	97 (33.2)	174 (24.5)
Improve getting out of bed, *n* (%)	192 (19.1)	77 (26.4)	115 (16.2)

**Table A4:** Reasons for Seeking Rehabilitation Services by Frailty Status Using the National Health and Aging Trends Study (NHATS), United States, 2015: Activities.

Activities	NHATS Total (*n* = 1003)	Frail (*n* = 292)	Non-Frail (*n* = 711)
Improve caring for self, *n* (%)	409 (40.8)	164 (56.2)	245 (34.5)
Improve household activities, *n* (%)	349 (34.8)	110 (37.7)	239 (33.6)
Improve working/volunteering, *n* (%)	95 (9.5)	20 (6.9)	75 (10.6)
Improve participating in activities, *n* (%)	169 (16.9)	51 (17.5)	118 (16.6)
Improve providing care, *n* (%)	46 (4.6)	11 (3.8)	35 (4.9)
